# Reassessing the Potential Activities of Plant CGI-58 Protein

**DOI:** 10.1371/journal.pone.0145806

**Published:** 2016-01-08

**Authors:** Abdallah Khatib, Yani Arhab, Assia Bentebibel, Abdelkarim Abousalham, Alexandre Noiriel

**Affiliations:** Institut de Chimie et de Biochimie Moléculaires et Supramoléculaires UMR 5246 CNRS, Organisation et Dynamique des Membranes Biologiques, Université Lyon 1, Villeurbanne, France; University of Graz, AUSTRIA

## Abstract

Comparative Gene Identification-58 (CGI-58) is a widespread protein found in animals and plants. This protein has been shown to participate in lipolysis in mice and humans by activating Adipose triglyceride lipase (ATGL), the initial enzyme responsible for the triacylglycerol (TAG) catabolism cascade. Human mutation of CGI-58 is the cause of Chanarin-Dorfman syndrome, an orphan disease characterized by a systemic accumulation of TAG which engenders tissue disorders. The CGI-58 protein has also been shown to participate in neutral lipid metabolism in plants and, in this case, a mutation again provokes TAG accumulation. Although its roles as an ATGL coactivator and in lipid metabolism are quite clear, the catalytic activity of CGI-58 is still in question. The acyltransferase activities of CGI-58 have been speculated about, reported or even dismissed and experimental evidence that CGI-58 expressed in *E*. *coli* possesses an unambiguous catalytic activity is still lacking. To address this problem, we developed a new set of plasmids and site-directed mutants to elucidate the *in vivo* effects of CGI-58 expression on lipid metabolism in *E*. *coli*. By analyzing the lipid composition in selected *E*. *coli* strains expressing CGI-58 proteins, and by reinvestigating enzymatic tests with adequate controls, we show here that recombinant plant CGI-58 has none of the proposed activities previously described. Recombinant plant and mouse CGI-58 both lack acyltransferase activity towards either lysophosphatidylglycerol or lysophosphatidic acid to form phosphatidylglycerol or phosphatidic acid and recombinant plant CGI-58 does not catalyze TAG or phospholipid hydrolysis. However, expression of recombinant plant CGI-58, but not mouse CGI-58, led to a decrease in phosphatidylglycerol in all strains of *E*. *coli* tested, and a mutation of the putative catalytic residues restored a wild-type phenotype. The potential activities of plant CGI-58 are subsequently discussed.

## Introduction

The α/β hydrolase-type protein Comparative Gene Identification-58 (CGI-58), also called ABDH5, is found in various organisms, such as mammals [1], insects [1], nematodes [2], birds [3] and plants [4], and it plays an important role in lipid metabolism, at least in mammals and in plants. In these two clades of evolution, *CGI-58* mutations provoke various neutral lipid disorders characterized, predominantly, by TAG accumulation in non-fat storing cells, such as the mesophyll cells of plants and the non-adipose tissues of mammals (skin, muscle cells etc.). Originally, human CGI-58 was described as being closely related to ICT1 (Increased Copper Tolerance 1) [5]. ICTI is a yeast protein identified by functional screening as being involved in tolerance to copper toxicity [6] and shown, by microarray analysis, to participate in isooctane tolerance [7].

In plants, a mutation in the gene for CGI-58 leads to an accumulation of TAG, with levels more than 10 times higher than in wild-type *Arabidopsis* leaves, a non-fat storing organ [4]. CGI-58 co-regulates lipid homeostasis, too, by interacting with Peroxisomal ABC Transporter (PXA1) [8], a protein that serves as a transporter of various substrates into peroxisomes and which is also required for fatty acid catabolism by β-oxidation [9]. In addition to its role as a lipid turnover regulator, it has been shown that CGI-58 also has an effect on polyamines, which have been described to be important molecules in growth and stress responses. In plants, CGI-58 regulates polyamine metabolism [10] by interacting with spermidine synthase 1 (SPDS1). Structurally, plant CGI-58 possesses at least two characteristic motifs. The first is a GXSXG motif found in lipases, containing a serine that, together with aspartate and histidine, is a member of a putative catalytic triad present in the α/β hydrolase-type family [1] to which plant CGI-58 belongs. The second one is a putative HX_4_D motif that is indispensable for acyltransferase activity [11]. An initial enzymatic study [12], published in 2009, showed that recombinant plant CGI-58 purified from *E*. *coli* possesses several activities, the main one being the catalysis of a lysophosphatidic acid (LPA) acylCoA acyltransferase (LPAAT) reaction. This study also reported hydrolase activities for TAG and phosphatidylcholine (PC) that were much less than 0.01% of the acyltransferase activity [12].

In mammals, the mutation of *CGI-58* is responsible for the Chanarin-Dorfman syndrome, a neutral lipid storage disease with ichthyosis (NLSDI), characterized by TAG accumulation within the epidermis, brain and liver [13]. The protein CGI-58 interacts with perilipin [14] and Adipose Triglyceride Lipase (ATGL) activating, by a factor of 20, the lipolytic activity of ATGL [15]. In addition, the mammalian CGI-58 protein possesses an acyltransferase motif, HX_4_D, and harbors a mutated GXNXG lipase motif, whereby a suspected inactive asparagine replaces the catalytic serine [1]. This protein has been shown to participate in TAG homeostasis, for example in cardiac and skeletal muscles [16]. Another independent enzymatic study of CGI-58 proteins has shown that mouse CGI-58 expressed in *E*. *coli* was also able, as the plant homolog, to catalyze an acyltransferase reaction [17]. However, the same authors demonstrated later that this activity was, in reality, an artifact due to a contaminant from *E*. *coli* that co-purified with the protein [18]. The contaminant was identified as plsC, the enzyme responsible for phosphatidic acid (PA) synthesis in *E*. *coli* through the transfer of the acyl group from acylCoA to LPA. It would be easy to imagine that the LPAAT activity, previously described in recombinant plant CGI-58 purified from *E*. *coli*, could originate from the same contaminant identified during the characterization of recombinant mouse CGI-58 purified from *E*. *coli*, raising reasonable doubts regarding the potential activity of recombinant plant CGI-58. More surprising results have emerged from a recent paper describing lysophosphatidylglycerol acylCoA acyltransferase (LPGAT) activity for human CGI-58, expressed in mammalian cells [19], questioning the putative activities and involvement of plant and mammalian CGI-58 in phospholipid synthesis.

Although the role of CGI-58 as a regulator of lipid metabolism is quite clear, its catalytic activity is still elusive and questionable. In order to reassess the putative activities of plant CGI-58, we tested the phenotype of several *E*. *coli* strains defective in lipid metabolism. We used new *in vivo* approaches to demonstrate that recombinant plant CGI-58 proteins are not able, to rescue an LPAAT mutant of *E*. *coli* nor to confer resistance to copper, which we have demonstrated has a toxic effect alleviated by moderate LPAAT activity. Likewise, these recombinant plant proteins did not show any detectable *in vitro* LPAAT activity, using purified protein obtained with an optimized protocol under native conditions [18]. Here, we report that the expression of plant CGI-58 in another *E*. *coli* strain deficient in phosphatidylglycerol (PG) synthesis could not restore the synthesis of PG in this genetic background, compared to an authentic PG synthase as prokaryotic pgsA. Also, more surprisingly, plant CGI-58 expression leads to a decrease in PG but not in phosphatidylethanolamine (PE) content, suggesting a role for CGI-58 in PG catabolism. Interestingly, a decrease in PG was also observed in all the tested *E*. *coli* strains expressing plant CGI-58 and we found that this effect was potentially due to the catalytic activity of CGI-58, via a mutation of the seryl residue within the GXSXG lipase motif at position 199. Using the decrease in PG as a phenotype, we tested the involvement of several residues, and we were able to show that only the histidyl, and not the aspartyl, residue belonging to the HX_4_D acyltransferase motif was implicated in the decrease of PG content.

## Materials and Methods

### Materials

The lipids dioleoylphosphatidylcholine (DOPC), 1-palmitoyl, 2-oleoylphosphatidylglycerol (POPG), 1-palmitoyl, 2-oleoylphosphatidylethanolamine and 1-oleoyllysoPG were purchased from Avanti Polar (Alabaster, Alabama, USA) and 1-oleoyllysophosphatidic acid, oleic acid, and triolein were purchased from Sigma Aldrich Chimie (Saint Quentin Fallavier, France). Strains MN7 [20], SM2-1 [21], and MG1655 were obtained from the *E*. *coli* Genetic Stock Center (Yale University). BL21(DE3) strain and plasmids pACYC184 and pACYC177 were obtained from New England Biolabs. Plasmid pAR1219 was from Sigma Aldrich Chimie. *Mus musculus CGI-58* (*MmCGI-58*) was a gift of Dr Zechner (University of Graz, Austria), and pET28a-*MmCGI-58*-12His (referred to as *MmCGI-58*) was a gift from Prof. Brasaemle (Rutgers, The State University of New Jersey, USA). The *Arabidopsis thaliana* full-length cDNA clone RAFL19-16-I19, coding *Arabidopsis* CGI-58 (AtCGI-58), was developed by the plant genome project at the RIKEN Genomic Sciences Center (Japan) [22,23]. All strains were grown at 37°C except MN7 and SM2-1, which were kept at 30°C. When necessary, LB plates contained 250 μM CuSO_4_, 1 mM ABTS and 1 μM IPTG. Antibiotic concentrations were 0.1 μg/μL for kanamycin, 0.02 μg/μL for chloramphenicol, 0.05 μg/μL for streptomycin, 0.1 μg/μL for ampicillin and 0.01 μg/μL for tetracycline. Pre-coated TLC-sheets ALUGRAM^®^ Xtra SIL G/UV_254_ (Macherey Nagel) were used for lipids analysis in *E*. *coli*. Silica Gel G & GF Preparative Uniplate (Analtech, Newark, USA) were used for tests involving radioactivity. *Thermomyces lanuginosus* lipase (TLL) was kindly provided by Dr S. Patkar (Novo-Nordisk, Copenhagen, Denmark). Phospholipase A_2_, from porcine pancreas, and monoclonal anti-polyHistidine coupled to peroxidase antibody were purchased from Sigma Aldrich Chimie. Radiolabeled lipids [dipalmitoyl-1-^14^C] phosphatidylcholine (111 mCi / mmol), [oleoyl-1-^14^C] oleoyl Coenzyme A (53.7 mCi / mmol) and [9,10-^3^H(N)] triolein (108 Ci / mmol) were all purchased from Perkin-Elmer.

### Enzymatic Activities

LPAAT, phospholipase and triglyceride lipase activities were measured under the same conditions as described in [12], with some modifications. Basically, LPAAT activity was measured, in the presence of 50 μM of lysoPA and 10 μM oleoylCoA (13 000 dpm / test) in a 50 mM potassium phosphate buffer, at pH 7.0 for 10 minutes, and in a final volume of 100 μL. Phospholipase activity was quantified for 40 minutes, in the presence of 100 μM of dipalmitoylPC (20 000 dpm / test), in a 50 mM Tris HCl buffer, at pH 7.5, with 2 mM DTT and 1 μg of proteins in a final volume of 100 μL. The reaction mixture was sonicated for 5 min before adding the enzyme. Triglyceride lipase activity was measured in the presence of 100 μM of triolein (100 000 dpm / test) and 100 μM of sodium taurocholate in a 50 mM Tris HCl buffer, at pH 7.5 for 40 minutes, with 1 μg of protein in a final volume of 100 μL. Again, the reaction mixture was sonicated for 5 min before adding the enzyme. For all tests, the temperature was set to 32°C. After stopping the reaction by adding 100 μL of 0.1 N HCl, lipid products were extracted, with the appropriate unlabeled substrates and expected unlabeled products as carriers using chloroform:methanol (2:1, v/v) and then separated by TLC on Silica Gel G & GF Preparative Uniplates with unlabeled, but identical products used as the reference.

Radiolabeled dipalmitoylPG (DPPG) was synthesized by transphosphatidylation from [^14^C]-dipalmitoylPC (DPPC) using phospholipase D (PLD) in the presence of glycerol. PC substrate, composed of 35.5 nmol of unlabeled DPPC and 4.5 nmol of [^14^C]-DPPC, was sonicated for 10 min to form micelles in 150 μL of buffer containing 0.83 mM SDS, 0.83 mM Triton X100, 20 mM CaCl_2_, 500 mM glycerol and 50 mM Tris-HCl, pH 8. 200 ng of purified recombinant *Vigna unguiculata* PLD [24] was added and the reaction mixture was incubated for 1 h at 30°C. The reaction was terminated by adding 100 μL of 0.1 N HCl and lipids were extracted with a mix of chloroform:methanol (2:1, v/v). The lipids were then separated by TLC using a mix of chloroform:methanol:acetic acid (65:25:10, v/v/v). [^14^C]-DPPG was scraped from the silica plate, re-dissolved in chloroform:methanol (2:1, v/v), filtered and then stored at -20°C until further use. Assuming that enzymatic conversions of labeled and unlabeled PC were equivalent, the specific activity of radiolabeled DPPG was then calculated as 250 dpm / pmol.

The putative PG acylhydrolase activity of recombinant CGI-58 protein was tested using either the purified truncated version of AtCGI-58 (AtCGI-58 Trc) or cell extracts of *E*. *coli* expressing AtCGI-58 Trc or the S199N mutant version. A reaction mixture containing 10 nmol of DOPG and 20 pmol of [^14^C]-DPPG (250 dpm/pmol) was dispersed, by sonication, for 5 min in a final volume of 100 μL of 50 mM Tris-HCl buffer, pH 7.5. Next, 7 μg of purified AtCGI-58 Trc or 10 μg of proteins from the cell extract were added and the reaction mixture was incubated, at 30°C, for 2 h for the purified fraction or 30 min for the cell extract. Reactions were terminated by adding 100 μL of 0.1 N HCl. Lipids were extracted with chloroform:methanol (2:1, v/v) and separated, by TLC, using a chloroform:methanol:acetic acid (65:25:10, v/v/v) solution. The area containing free fatty acids was scraped and then quantified by scintillation counting.

### Cloning

cDNAs coding for MmCGI-58 and for full-length (AtCGI-58 FL) or truncated AtCGI-58 (AtCGI-58 Trc) were amplified by PCR with primers 01 and 02, 03 and 04, 03 and 05, respectively. The primers are listed in Table 1. All constructs harbor an *NdeI* site and a sequence coding for a 6-Histidine tag at the 5’ end, together with a *NotI* restriction site at the 3’ end. PCR products were digested with NotI and NdeI, purified with the QIAquick PCR Purification Kit (QIagen, Courtaboeuf, France), and then ligated into the pET28b plasmid (Novagen, Merck KGaA, Darmstadt, Germany) digested by the same restriction enzymes. CotA was amplified with primers 18 and 19, harboring a *PciI* and a *NotI* site, respectively, and cloned in the same way except that PciI was used instead of NdeI for digesting the PCR product. All constructs were verified by sequencing (Eurofins MWG, Ebersberg, Germany).

**Table 1:** List of primers used. Restriction sites are underlined, start codons are in bold, and mutated bases for site-directed mutagenesis are italicized.

Primer name	Target	Sequence 5→3’
01	MmCGI-58 Fwd	CAGTCAAC**ATG**TTAGCGATGGCGGCG
02	MmCGI-58 Rev	TGCTGCGGCCGCTCAGTCTACTGTGTGGC
03	AtCGI-58 FL Fwd	GTCACAT**ATG**AACTTGAGCCGTTTTGCTT
04	AtCGI-58 FL and Trc Rev	TGCTGCGGCCGCCTAAACCAATCGTAGACC
05	AtCGI-58 Trc Fwd	GTCACAT**ATG**AAATCAAGATGGAAAATTTTGTG
06	TetC Fwd	ATGAAATCTAACAATGCGCTCATC
07	T7RNA polymerase Rev	GTGCAATCATCTTAGGGAGTA
08	plsC Fwd	TCACC**ATG**GTATATATCTTTCGTCTTATTATTACCG
09	plsC Rev	TGCTGCGGCCGCTTAAACTTTTCCGGCGGCTTC
10	pgsA Fwd	TCAAC**ATG**TCATTTAATATCCCTACGTTGCTTAC
11	pgsA Rev	TGCTGCGGCCGCTCACTGATCAAGCAAATCTGCA
12	AtCGI-58 Trc S199N Fwd	CAT*AAT*TTTGGAGGCTATGTTGCTAAA
13	AtCGI-58 Trc S199N Rev	ATAGCCTCCAAA*ATT*ATGTCCTAATAG
14	AtCGI-58 Trc H379A Fwd	CCACAGGGTGGT*GCT*TTTGTGTTCATA
15	AtCGI-58 Trc H379A Rev	TATGAACACAAA*AGC*ACCACCCTGTGG
16	AtCGI-58 Trc D384A Fwd	TTTGTGTTCATA*GCC*AACCCAATTGGT
17	AtCGI-58 Trc D384A Rev	ACCAATTGGGTT*GGC*TATGAACACAAA
18	CotA Fwd	CAGTCAAC**ATG**TCACTTGAAAAATTTGTGGATGCT
19	CotA Rev	GATGCTGCGGCCGCTTATTTATGGGGATCAGTTATATCC

The pYAT7 plasmid (8694 bp) (accession number KT970713) was constructed by excising the *lacUV5-T7 RNA polymerase* (*T7RP*) fragment from the pAR1219 plasmid with BamHI, then cloning it into the *BamH1* site of the plasmid pACYC184. The orientation of the insert was confirmed by sequencing the construct with primer 06 (Table 1), located at the 5’ end of *tcR*. The sequence between the *lacI* promoter and the *T7RP* open reading frame was also obtained from sequencing with the reverse primer 07 (Table 1), located at the 5’ end of *T7RP*. The exact size of the plasmid was calculated from the aforementioned sequences obtained from sequencing reactions, using information provided by [25], and from the pACYC184 sequence (accession number X06403.1).

In order to generate pAKT7 (accession number KT970712), a vector conferring resistance to ampicillin and allowing the inducible expression of the T7RNA polymerase, the pYAT7 plasmid was digested by HindIII and SalI and the pACYC177 plasmid was digested by HindIII and XhoI. Since *SalI* and *XhoI* are compatible sites, the fragment containing the *T7RNA polymerase* sequence, roughly 5000 bp long and resulting from digested pYAT7, was then ligated into the kanamycin resistance cassette of the pACYC177 plasmid.

The gene coding for plsC was amplified from the genome of the *E*. *coli* strain BL21(DE3), by PCR, using primers 08 and 09 (Table 1) or from the strain SM2-1 for the plsC*G39E variant. Amplification was performed using KOD polymerase (Novagen). This strategy generates a construct with the restriction sites *NcoI* and *NotI* at the 5' and 3' ends, respectively. The PCR product was then ligated into the dephosphorylated pET28b(+) vector digested with NcoI and NotI. The gene coding for pgsA was amplified from the *E*. *coli* MG1655 strain, by PCR, using primers 10 and 11 (Table 1) and Q5 polymerase (Biolabs, Evry, France). The fragment generated, containing a *PciI* and a *NotI* restriction site at the 5’ and 3’ ends, respectively, was further ligated into the pET28b(+) plasmid digested by NcoI and NotI. Both constructs were verified by sequencing, as described above.

### Expression

Plasmids were transferred either into SM2-1 and MN7 strains previously transformed with the pYAT7 vector or directly into the BL21(DE3) strain. All transformations were carried out by electroporation using a Bio-Rad Micropulser. The resulting transformants were grown on LB plates with appropriate antibiotics at 37°C for the BL21(DE3) strain or at 30°C for the others.

For protein expressions, clones were grown overnight in LB medium, containing appropriate antibiotics, and at the temperature described previously. Cultures were then diluted 10 times and grown until the OD_600 nm_ reached 0.6–0.8. Induction, for 5 h, was initiated by the addition of 1 mM IPTG.

For SDS-PAGE analysis, the conditions were identical except that IPTG was added for 3 h at 30°C. The expression level was tested by loading the total proteins extracted from an equivalent of 100 μL of culture at an OD_600 nm_ of 1.

To prepare cell extracts, containing soluble AtCGI-58, MmCGI-58 or plsC, 100 mL of culture was harvested by centrifugation. The pellet was then incubated with 1 mg/mL of lysozyme, for 30 min at 4°C, in lysis buffer containing 50 mM NaH_2_PO_4_ and 300 mM NaCl at pH 7.4. Cells were disrupted by probe sonication. Cell debris were then removed, by centrifugation at 10 000 x *g* for 20 min, and the soluble fraction was used for enzymatic tests or purification.

### Purification

The soluble fraction, obtained as described above, was incubated with TALON^®^ Metal Affinity Resin (Clontech). An extract containing AtCGI-58 was incubated, for 3 h at 4°C, in a buffer composed of 50 mM NaH_2_PO_4_, 300 mM NaCl, and 20 mM imidazole at pH 7.4. An extract containing MmCGI-58 was incubated overnight at 4°C in a buffer consisting of 50 mM NaH_2_PO_4_, 100 mM KCl, 30 mM imidazole, 1 mM DTT and 10% glycerol at pH 7.5 (Prof. Brasaemle, Rutgers, The State University of New Jersey, USA; personal communication). Resin was then recovered, by centrifugation, and washed with the same buffers as described previously except that the imidazole concentration was increased to 25 mM for AtCGI-58 and to 100 mM for MmCGI-58 purification. Any bound proteins were then eluted with the same buffers, containing 250 mM imidazole, and desalted on PD-10 columns equilibrated in 50 mM Tris-HCl, pH 8, plus 10% (v/v) glycerol. The concentration of purified protein was estimated by measuring absorbance at 280 nm using a Nanodrop spectrophotometer (Thermo Fisher Scientific). The authenticity of MmCGI-58 was checked by mass spectrometry following tryptic digestion (Centre d’Analyses Protéomiques IMM-CNRS, Marseille, France). For plant CGI-58, proteins were transferred, after SDS-PAGE, onto nitrocellulose membranes and the presence of the N-terminal His-tag was verified by development with a monoclonal anti-polyHistidine-peroxidase antibody.

### Mutagenesis

AtCGI-58 mutations were generated using the GeneArt^®^ Site-Directed Mutagenesis PLUS system from Invitrogen. The cDNA coding for the truncated version of AtCGI-58 and cloned into the pET28b vector was used as a template. Primers, described in Table 1, were used to substitute H379 and D384 with alanyl residues, and S199 in the lipase motif of AtCGI-58 was replaced by an asparaginyl residue, as found in MmCGI-58.

### Lipid extraction

Total lipids were extracted using the method of Bligh and Dyer, as modified by Ames [26,27]. *E*. *coli* cells were induced in 25 mL of culture, as previously described for protein expression except that induction was reduced to 4 hours. Cells were then harvested with a 4 000 x *g* centrifugation for 10 min, the pellet was re-suspended in 0.8 mL of PBS, and 2 mL of methanol and 1 mL of chloroform were added with thorough mixing. After 1 h at room temperature, 1 mL of chloroform and 1 mL of PBS solution were added and then the phases were thoroughly mixed. After a brief low-speed centrifugation, the resulting organic phase was carefully removed, dried under N_2_, re-suspended in a small volume of chloroform and then analyzed for individual phospholipids. Two-dimensional thin layer chromatography, on pre-coated TLC-sheets ALUGRAM^®^ Xtra SIL G/UV_254_ (Macherey Nagel), was used to separate the different lipids. For the first dimension, a solution of chloroform / methanol / water (65:25:4, v/v/v) was used and then a chloroform / methanol / acetic acid (65:25:10, v/v/v) solution was employed for the second dimension. After drying the plates, lipids were visualized by exposure to iodine vapor.

### Lipid quantification

PG and PE were quantified by LC/MS/MS. Total lipids extracted as mentioned previously, were re-suspended in 2.5 mL of methanol. An aliquot (200 μL) of each sample was filtrated, dried under N_2_, re-suspended in 200 μL of acetonitrile / methanol / chloroform (18:1:1, v/v/v) and then 10 μL of this sample were injected for analysis. The LC system consists of two LC-30AD binary pumps, a SIL-30AC autosampler and a CTO-20AC column oven (Shimadzu). Samples were separated on a CORTECS HILIC column 2.7μm 4.6x150 mm from Waters.

The gradient elution used eluents A and B, where A was 95/5 acetonitrile/water with 10 mM ammonium formate and B was 50/50 acetonitrile/water with 10 mM ammonium formate. The column was equilibrated for 2 min with 0% of B prior to injection of the sample. The percentage of B was held constant for 2 min and it was then linearly increased from 0 to 50% over a period of 8 min. The flow rate was set to 0.5 mL/min and the column temperature to 30°C. The QTRAP 4500 mass spectrometer, equipped with a heated ESI source (ABSciex) was operated in positive mode. Nitrogen was used as both the sheath and the curtain gas and it was set to 20 and 40 psi, respectively. The sprayer voltage was set at 4500 V and the collision gas at medium. The vaporized temperature was set at 350°C.

For the neutral loss scan experiments, the MS/MS scan consists of a search for neutral loss of 189 Da for PG analysis, and 141 Da for PE. The mass range was from *m/z* 600 to 1000 for PG and PE, and the scan rate was 1000 Da/s for each experiment. The declustering potential was set to 95, entrance potential to 10, collision energy to 37 and collision cell exit potential to 13 for all the experiments. The quantity of PG and PE was calculated based on a 28:0 PG and 36:2 PE standard curve, respectively.

### Statistical analysis

Data are expressed as means ± S.D. Statistical significance was determined by the Student’s unpaired t-test (two-tailed). Samples were considered to be significantly different for *P* <0.05 (*), *P*< 0.01 (**), and *P* < 0.001 (***).

## Results and Discussion

### Development of a new set of plasmids allowing *E*. *coli* expression of genes cloned into pET plasmids

**Fig 1:**
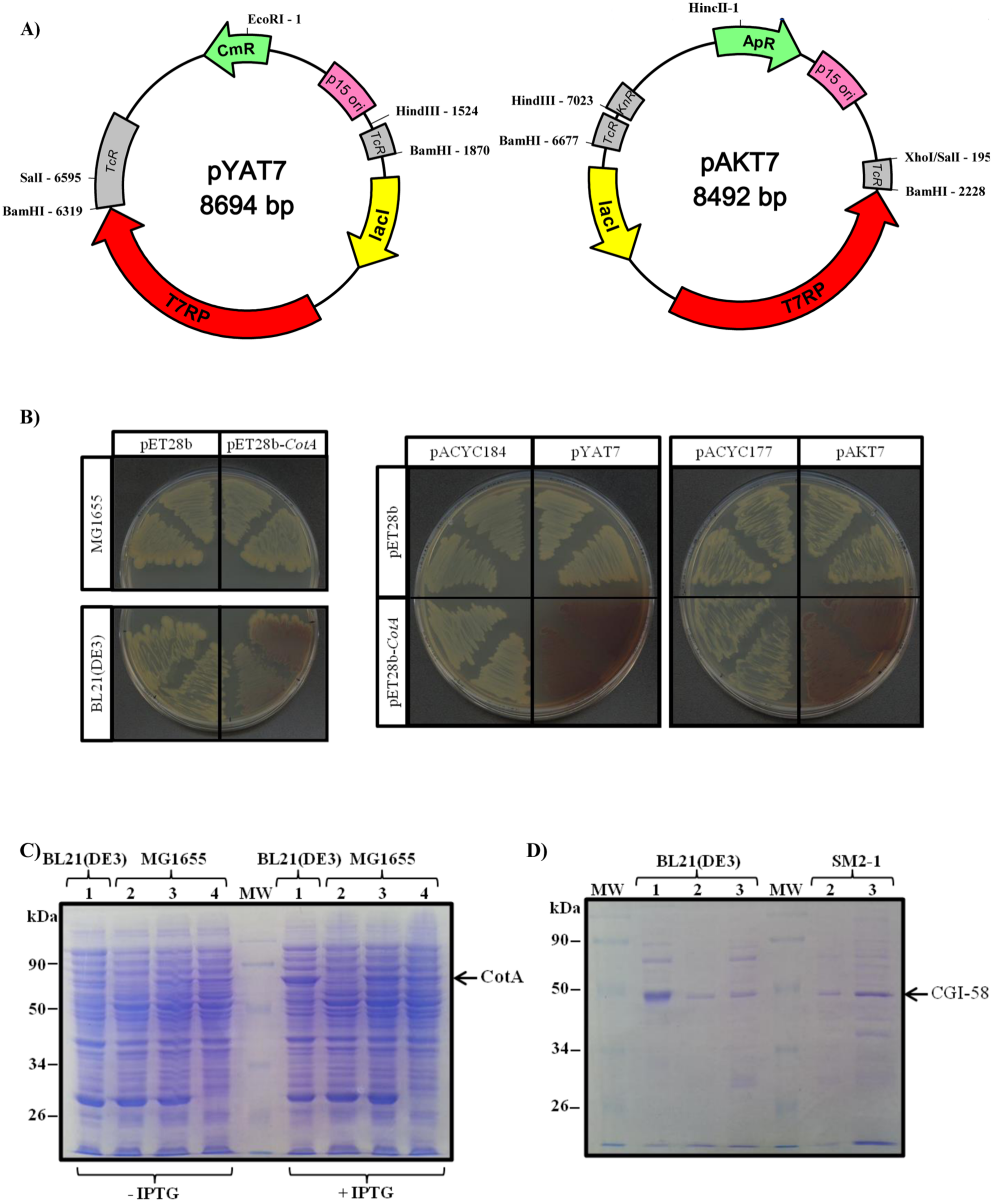
A new set of plasmids containing *T7 RNA polymerase* and with a replication origin compatible with the pET plasmid series, allowing expression and purification of recombinant proteins in *E*. *coli*. A) Maps of vectors pYAT7 and pAKT7 containing *T7 RNA polymerase* cloned into plasmids harboring the p15A origin of replication. Left panel: pYAT7 plasmid with *T7RP* cloned into the *BamHI* site of the pACYC184 backbone, thus conferring resistance to chloramphenicol. Right panel: pAKT7 plasmid with *T7RP* cloned into the *HindIII-SalI* sites of the pACYC177 backbone, thus conferring resistance to ampicillin. Active and inactive antibiotic resistance genes are shown in green and in grey, respectively. Remarkable restriction sites are shown. B) Functional use of pYAT7 and pAKT7 plasmids for expressing the recombinant CotA protein in MG1655, a T7RP deficient *E*. *coli* strain. Left panel: coloration due to ABTS oxidation in different *E*. *coli* genetic backgrounds necessitates both the presence of CotA and T7RP. MG1655 or BL21(DE3) strains were either transformed with void pET28b or with pET28b-*CotA*. Central and right panels: functional expression of CotA in MG1655 strain transformed with either pYAT7, pAKT7 plasmids or native pACYC184 or pACYC177 plasmids. LB plates contain 250 μM CuSO_4_, 1 mM ABTS and 1 μM IPTG. All pictures were taken 3 days after streaking. C) Functional expression of CotA cloned into pYAT7 and pAKT7 plasmids with analysis by SDS-PAGE of total proteins extracted from the recombinant strain. Extracts were analyzed before or after a 3 h induction with IPTG. Lane 1: strain BL21(DE3) transformed with pET28b-*CotA*. Lane 2 and 3: strain MG1655 transformed with pYAT7 and either void plasmid (lane 2) or pET28b-*CotA* (lane 3). Lane 4: strain MG1655 transformed with pAKT7 and pET28b-*CotA*. The expected size of CotA (58.5 kDa) is indicated. D) Analysis by SDS-PAGE of purified CGI-58, from plant or mouse, expressed either in BL21(DE3) strain or SM2-1 and transformed with pYAT7. 5 μg of recombinant MmCGI-58 and 2 μg of recombinant plant CGI-58 were loaded onto each lane. Lane 1: MmCGI-58 (42.0 kDa), Lane 2: truncated version of AtCGI-58 (44.5 kDa), Lane 3: full-length version of AtCGI-58 (47.8 kDa). The authenticity of purified recombinant MmCGI-58 was verified by mass spectrometry after tryptic digestion, and for recombinant plant CGI-58 a Western-blot with monoclonal Anti-polyHistidine antibody was performed.

Constructs designed for protein overexpression in *E*. *coli* are frequently made into pET plasmids (Novagen), cloned 3’ to the T7 phage promoter. These pET plasmids confer resistance to either kanamycin or ampicillin and have a pBM1 origin of replication. However, although these plasmids are useful in BL21(DE3) strains hosting chromosomal *T7RP*, they are of no use in other genetic backgrounds lacking *T7RP*. T7RP is indispensable for T7 promoter recognition, especially in *E*. *coli* mutants that are potent tools for complementation and for tests on proteins of unknown function. Expressing proteins in these valuable strains necessitates fastidious constructs and sub-cloning into other plasmids with a different promoter. In order to bypass these problems, we decided to clone the *T7RP* into plasmids that had a compatible origin of replication, such as p15A and, at the same time, provide a different antibiotic resistance. Hence, we excised *lacI* and *T7RP* from the pAR1219 vector with BamHI and sub-cloned this fragment into the *tet* gene of a pACYC184 plasmid, previously digested with BamHI. In this way, we abolished the tetracycline resistance and then generated the pYAT7 plasmid with resistance to chloramphenicol only [Fig 1A]. *lacI* and *T7RP* were then excised from pYAT7, with SalI and HindIII, and the fragment was sub-cloned into the kanamycin resistance gene of the pACYC177 plasmid digested with XhoI and HindIII. *SalI* and *XhoI* sites have compatible ends and we then formed the pAKT7 plasmid, in this case conferring resistance to ampicillin [Fig 1A].

Recombinant laccase CotA has been previously shown to colorize the medium when successfully expressed in *E*. *coli* [28]. To prove the effectiveness of the two novel plasmids we had constructed, we transformed the MG1655 strain deficient in T7RP with one of these two plasmids and *CotA* was cloned into the pET28b. When transformed with pET28b-*CotA*, coloration due to ABTS oxidation by CotA was observed only in the BL21(DE3) strain containing a chromosomal *T7RP* [Fig 1B, left panel], and coloration was absent in MG1655, which does not contain *T7RP*. Coloration could be observed again when MG1655 was transformed with the construct pET28b-*CotA*, and either pYAT7 or pAKT7, but not with the native plasmids pACY177 or pACYC184 [Fig 1B, central and right panels]. This demonstrates that T7RP was both functionally expressed and allowed the expression of the recombinant protein whose corresponding gene was cloned into pET28b. To confirm that the level of expression was comparable to that found in BL21(DE3), total proteins from these transformants, grown with or without IPTG, were extracted and separated on SDS-PAGE [Fig 1C, left part]. As expected, the expression of recombinant CotA (theoretical molecular weight 58.5 kDa) was shown to be IPTG dependent and its expression level was quite similar, though slightly lower, in the common strain MG1655, transformed with either pYAT7 or pAKT7, compared to commercial BL21(DE3). Both plasmids could now be used in any strain deficient in T7RP in order to express *in vivo* genes cloned under the T7 promoter. They would be powerful tools for overexpressing recombinant proteins and to test for functional complementation with the same construct cloned into pET plasmids.

A comparison of plant and mammalian CGI-58 primary sequences reveals that they differ, in particular with regard to their N-terminal regions, as plant CGI-58 typically has an extension of about thirty residues. We searched for a CGI-58 homolog from different plant species of all taxa, from Mosses through to Eudicots. It was quite remarkable that the sequence homology begins 50 residues after the first methionine, suggesting divergent evolution of the N-terminal region and a strong conservation of the central and C-terminal parts [S1 Fig]. Bioinformatic software, such as TargetP [29], projects a transit peptide, either chloroplastic or mitochondrial, for CGI-58 for 24 out of the 28 plant sequences examined. Admittedly, plant CGI-58 was described as being cytosolic [4], but it was also found on the surface of peroxisomes [8]. Consequently, to avoid a probable misfolding of the protein expressed in *E*. *coli* due to a potential uncleaved transit or targeting peptide, we decided to test two versions of the *Arabidopsis* protein: full-length and one truncated at position 43.

We expressed the mouse CGI-58, plus the truncated and the full-length versions of CGI-58 from *A*. *thaliana*, in either BL21(DE3) or SM2-1 strains, the latter being deficient in *T7RP*, but previously transformed with a pYAT7 plasmid. Recombinant proteins were purified under native conditions according to published [18] and unpublished (Prof. Brasaemle, personal communication) protocols for mouse CGI-58 and an optimized protocol for plant CGI-58 (this study). Fractions were considerably enriched in target proteins, compared to crude extracts, and we were able to purify enough recombinant plant protein due to the expression capacity of the pYAT7 vector (24 to 36 micrograms of protein were purified per liter of culture). After SDS-PAGE, the fractions appeared to be enriched principally in the targeted protein [Fig 1D].

### Reassessing the phosphatidic acid synthase activity of recombinant plant CGI-58

**Fig 2:**
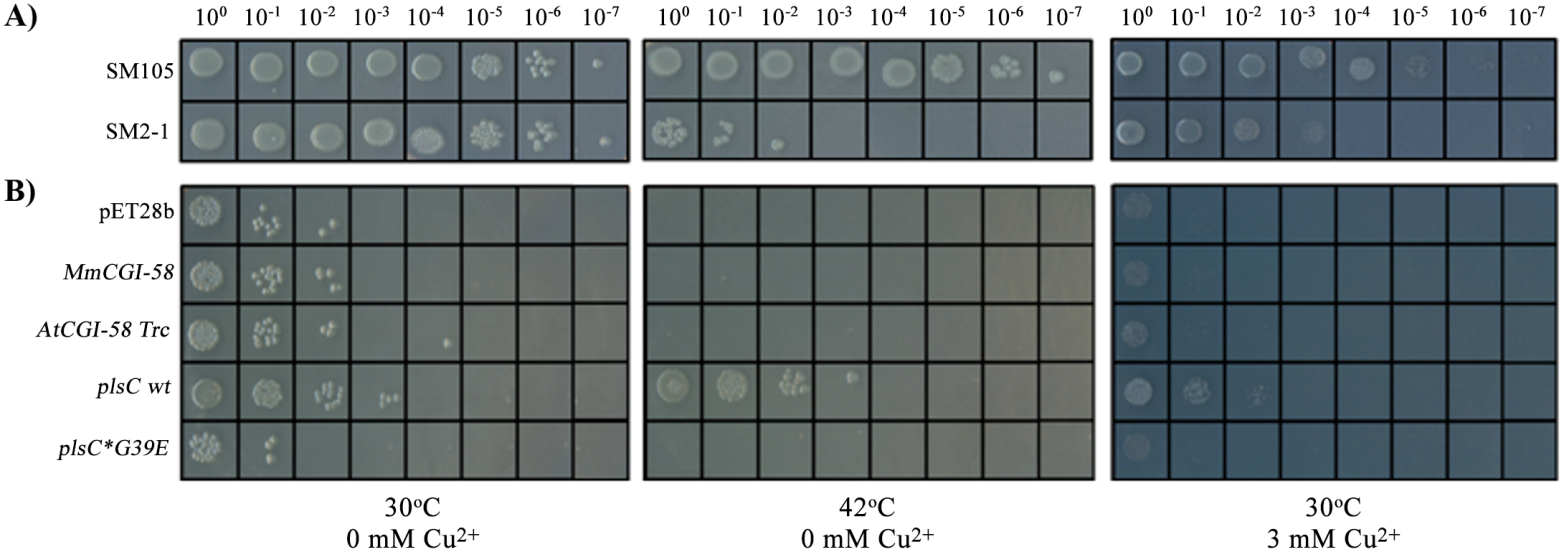
Absence of lysphosphatidic acid acylCoA acyltransferase activity of plant CGI-58 protein shown by functional complementation. A) Phenotype of SM2-1 and SM105 strains, grown at 30 or 42°C in the presence or absence of 3 mM of copper sulfate after serial dilution. B) Serial dilutions of the SM2-1 strain containing the pYAT7 plasmid and transformed with *CGI-58* constructs or controls: void pET28b vector, *MmCG-58*, *AtCGI-58 Trc*, *plsC wt*, or plsC*G39E. Liquid cultures were incubated for 4 hours with 100 μM IPTG prior to dilution and plating. LB plates contained, in addition to antibiotics, 100 μM IPTG. All pictures were taken 2 days after plating.

Like the mammalian protein, recombinant plant CGI-58 has been previously described as catalyzing an LPAAT reaction. However, activity of the mammalian protein was recently shown to be due to an *E*. *coli* contaminant that co-purified with the protein [18]. In order to reevaluate the plant enzymatic activity, we tried to complement the SM2-1 strain deficient in LPAAT activity as a result of the *plsC* mutation. Compared to the wild-type strain SM105, this *plsC* mutant is unable to grow at 42°C [Fig 2A]. Complementing this strain with an enzyme catalyzing the synthesis of PA restores growth at this permissive temperature [30,31,32,33]. We co-transformed *CGI-58* constructs, cloned into the pET28b vector, with the pYAT7 plasmid in the SM2-1 strain and then performed a serial dilution of the cultures. The void plasmid was used as a first negative control. As a positive control, we cloned *plsC* from BL21(DE3) and as a second negative control we used plsC*G39E from SM2-1. This mutated gene coding for a G39E variant of plsC is responsible for the temperature sensitivity observed at 42°C [34]. The expression of different *CGI-58* constructs did not restore growth at 42°C in the way that the expression of authentic *plsC* did, whereas expression of the plsC variant G39E showed no difference compared to the void control [Fig 2B]. Clones expressing AtCGI-58 FL behaved in the same way as those transformed with the truncated version of the protein (data not shown). This demonstrates that an active LPAAT, such as plsC, can be detected in this expression system and that strains transformed with different CGI-58s behave as negative controls.

CGI-58 was previously identified by its homology with yeast ICT1 (Increased Copper Tolerance 1) [5]. Since an *ict1* mutation causes copper sensitivity [6], one could hypothesize that CGI-58 proteins participate in copper tolerance or detoxification too. We first decided to examine the sensitivity of strain SM2-1 to copper. Interestingly, this strain deficient in LPAAT activity is considerably more sensitive to copper than the SM105 wild-type strain, proving that plsC confers an advantage in regards to copper toxicity and that lipid composition is related to copper permeability [Fig 2A]. When different *CGI-58* constructs were expressed in the presence of 3 mM of copper, no difference in growth could be observed compared to the negative controls under these conditions. AtCGI-58 FL and murine CGI-58 with 6 (data not shown) or 12 His-tag behaved in the same manner.

However, a clone expressing *plsC* was more resistant to copper than the control and a clone expressing the mutated version of plsC was comparable in behavior to the void control [Fig 2B]. Under these conditions, no complementation of this strain could be observed with different CGI-58 constructs, especially with those of plants. The same results were obtained when transformants were grown in liquid medium with 50 μM of copper (data not shown). An involvement of PA synthesis has already been described in vancomycin resistance in *E*. *coli* [35], but this is the first report, to our knowledge, of a direct link between PA synthesis and copper sensitivity.

Since CGI-58 is described as being located in the cytosol [4] and plsC has been shown to be membrane-bound by the presence of a putative anchor in its N-terminal region [36], allowing for co-localization of the protein and its substrates, a chimera was made of the first 40 amino acids of plsC fused to the N-terminal region of mouse CGI-58. No difference under the temperature and copper toxicity conditions described above was observed compared to native CGI-58 and negative controls (data not shown). This corroborates the results obtained by other researchers [18]. We observed, after SDS-PAGE analysis of proteins purified from membrane fractions, that mouse CGI-58 is already partially membrane-bound in *E*. *coli* and that adding the first 40 amino acids of plsC leads to a similar amount of mouse CGI-58 protein in the membrane fraction (data not shown).

**Fig 3:**
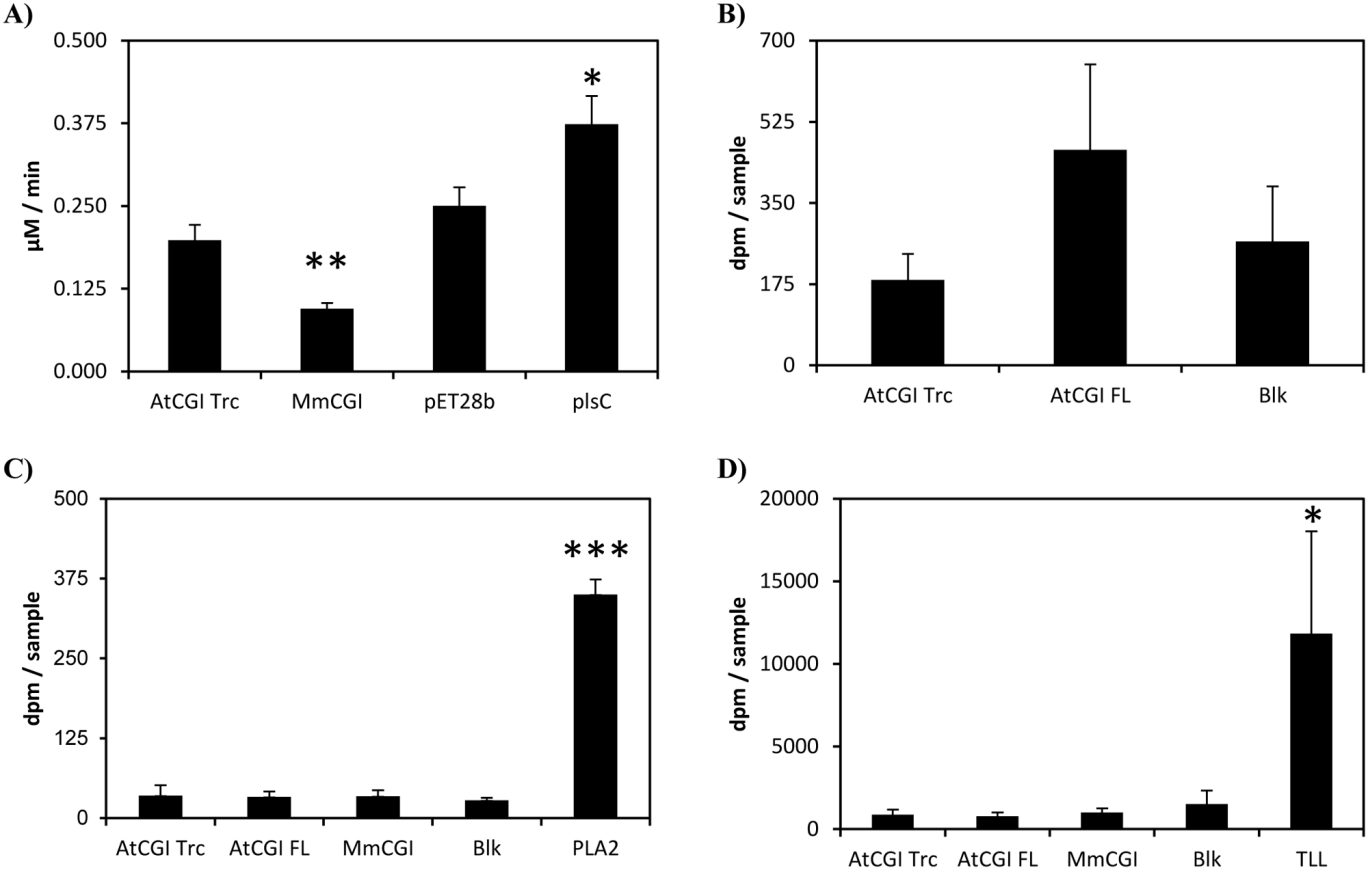
Reassessing the potential activities of recombinant plant CGI-58 using enzymatic tests. A) Measurement of PA formation, through LPAAT activity, using 5 μg of protein from a crude extract of SM2-1 cells transformed either with the void pET28b vector, or the truncated version of *AtCGI-58*, or *MmCGI*-58 or *plsC*. Activities were corrected from the blank values obtained with samples tested in the absence of protein; blank values represent less than 10% of the dpm counted with other samples. Values ± SD are the mean of three independent measurements. *P<0.05, ** *P*<0.01 (*vs* pET28b). The experiment was repeated with similar results. B) Measurement of total dpm found in the PA fraction after TLC separation with purified recombinant plant CGI-58. LPAAT activity was tested with samples incubated for 10 min in the presence of 1 μg of a purified full-length or truncated version of *AtCGI-58* expressed in the SM2-1 strain. Blanks (Blk) were prepared with no protein incubation. Values ± SD are the mean of three independent measures. The experiment was repeated with similar results. C) Measurement of the phospholipase activity of purified recombinant plant CGI-58. (D) Measurement of the triglyceride lipase activity of purified recombinant plant CGI-58. For both figures, the total dpm counted in the free fatty acid fraction after TLC separation is shown. Samples were incubated for 40 min in the presence of 1 μg of a purified full-length or truncated version of *AtCGI-58* expressed in the SM2-1 strain. Porcine pancreatic phospholipase A_2_ (1 ng) and triglyceride lipase from *Thermomyces lanuginosus* (TLL) (100 ng) were used as positive controls. Blanks (Blk) were prepared with no protein incubation. Values ± SD are the mean of three independent measures. * *P*<0.05, *** *P*<0.001 (*vs* blank). The experiment was repeated with similar results.

Considering the possibility that the plant enzyme and its substrates are not close enough together, *in vivo*, to permit a catalytic reaction, we next tried to measure *in vitro* LPAAT activity with a purified protein. Our first attempts to measure the activity, as described previously [12], were unsuccessful. As detailed by Ghosh *et al*., recombinant plant CGI-58 was purified from inclusion bodies, under denaturing conditions with 6 M urea, and tested without any proper refolding step, but it is possible that the refolding was immediate when the protein was mixed in the reaction buffer. Consequently, we tried to measure LPAAT activity using recombinant plant CGI-58 purified under native conditions from BL21(DE3) [Fig 1D] and we were able to detect, by LC-MS, a weak PA synthesis over time. However, at the same time as we were performing this experiment, McMahon *et al*. showed that the previously described LPAAT activity for mouse CGI-58 was emanating, in fact, from *E*. *coli* plsC that co-purifies with CGI-58 [18]. We decided to continue with the expression of CGI-58 and plsC in SM2-1 to minimize proven contamination during purification from plsC. We tested crude extracts and proteins purified under native conditions for LPAAT activity. Although an increase in activity was measured in a crude extract from SM2-1 cells transformed with *plsC*, no increase in LPAAT activity was observed in any other samples tested [Fig 3A]. The activity in samples expressing recombinant plant CGI-58 was the same as that of the control and, notably, no activity could be detected with recombinant plant purified protein compared to the mock sample [Fig 3B]. Interestingly, activity in the crude extracts of samples expressing MmCGI-58 was consistently and repeatedly half of that observed in the control, suggesting that MmCGI-58 could modulate plsC activity directly or indirectly and it could have an effect on lipid metabolism in *E*. *coli*.

We tested the phospholipase and TAG lipase activities of recombinant plant CGI-58 purified under native conditions with porcine pancreatic PLA_2_ and TLL, two well-known lipolytic enzymes, as positive controls for each activity. Although fatty acids were freed from PC and TAG by PLA_2_ and TLL, respectively, no free fatty acids could be detected as a result of recombinant plant CGI-58 incubation, as shown in Fig 3C and 3D. No differences were observed between all the samples tested and, in particular, the radioactivity measured with purified recombinant plant CGI-58 was the same as the background measured in the blank prepared without any enzyme. Whilst the LPAAT activity, previously described for recombinant plant CGI-58 [12], may have resulted from plsC contamination, the absence of phospholipase and TAG lipase activities with our recombinant plant CGI-58 proteins, purified under native conditions in our study, is puzzling. These enzymatic activities have been described [37,38], or suspected [39], in *E*. *coli*, but, to our knowledge, no protein has been identified or characterized so far, rendering the hypothesis of another contamination improbable but still possible. However, recombinant plant CGI-58 was previously purified from urea-solubilized inclusion bodies [12], therefore it is still possible that the refolding step in an urea-free buffer leads to conformational changes affecting the enzymatic properties of the protein. More probably, traces of urea resulting from purification under denaturating conditions may have increased these weak enzymatic activities, dilute denaturants, such as urea, being well-known activators of catalytic activities [40,41,42,43].

### Assaying the phosphatidylglycerol synthase activity of recombinant plant CGI-58

Since it has recently been suggested that mammalian CGI-58 catalyzes PG synthesis [19], we decided to test the activity of plant and mouse CGI-58 in *E*. *coli* with functional complementation. We used the MN7 strain, deficient in PG synthesis, where there is a mutation in *pgsA*, which provokes a phenotype with temperature-sensitivity that can be alleviated by expressing a phosphatidylglycerate synthase [20,44]. Even though *pgsA* encodes a CDPdiacylglycerol: *sn*-glycerol-3-phosphate phosphatidyltransferase and not a lysophosphatidylglycerol acylCoA acyltransferase, lysoPG is still found in *E*. *coli* [45,46]. Consequently, a lysoPG acyltransferase, or any enzyme catalyzing the formation of PG from intermediates present in *E*. *coli*, may overcome this lethal phenotype. When expressed in the MN7 strain with the pYAT7 plasmid, which allows the expression of genes cloned into the pET28b plasmid, none of the clones expressing the different CGI-58 constructs could grow at 42°C (data not shown). This demonstrates that, under these conditions, CGI-58s seem not to be involved in PG synthesis *in vivo*.

**Fig 4:**
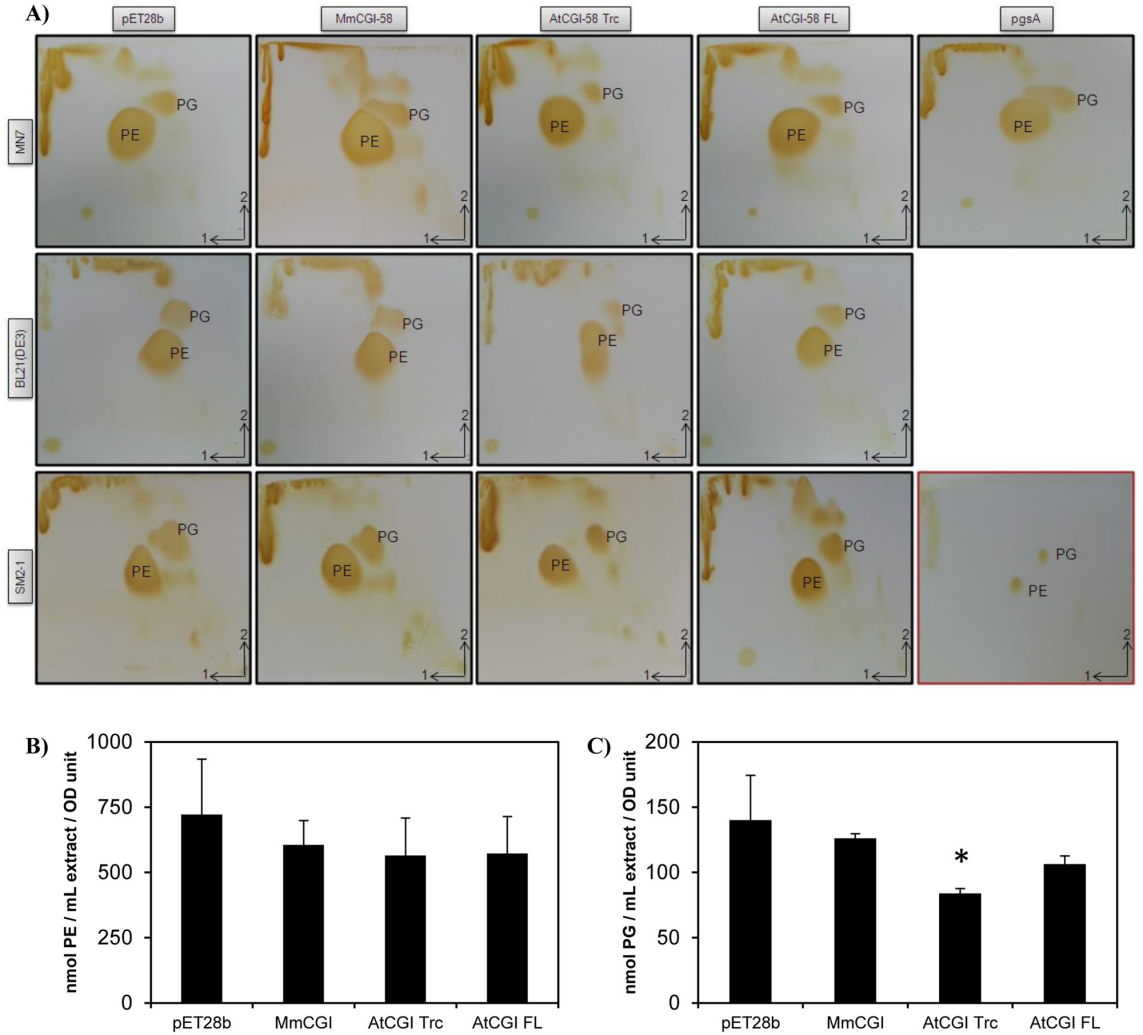
Analysis of phospholipids extracted from strains transformed with different versions of *CGI-58*. Strain MN7 deficient in PG synthesis [20], wt strain BL21(DE3) and strain SM2-1 deficient in PA synthesis [21] were all transformed with either the void plasmid, the *CGI-58* from mouse, or the full-length or truncated version from plant *CGI-58*. *pgsA* was also used as a positive control to restore PG synthesis in MN7. MN7 and SM2-1 strains had been previously transformed with pYAT7. A) TLC analysis of total lipids extracted after a 4 h-induction of transformants by 1 mM IPTG, then separated by 2-D TLC with, first, chloroform/methanol/water (65:25:4, v/v/v), and then with chloroform/methanol/acetic acid (65:25:10, v/v/v). After drying the plates, the lipids were visualized by exposure to diiodine vapor. TLC with authentic PE and PG standards is shown in the bottom right-hand corner (red frame). Each experiment was repeated at least thrice. B) Measurement of PE content of SM2-1 transformants determined by LC/MS/MS. Total lipids were extracted as above and analyzed. The quantities of PE were calculated based on a 36:2 PE standard curve and normalized with the OD_600nm_ values of the liquid cultures after a 4 h-induction by IPTG. Values ± SD are the mean of three independent clones. C) PG content in SM2-1 transformants determined by LC/MS/MS. Total lipids were extracted as above and analyzed. The quantities of PG were calculated based on a 28:0 PG standard curve and normalized with the OD values as above. Values ± SD are the mean of three independent clones. * *P*<0.05, (*vs* pET28b).

However, expressing *pgsA*, cloned into the pET28 plasmid in the MN7 strain, did not give a clear positive control at 42°C, potentially due to the toxicity of PGSA expression or activity that we observed at 30°C with this particular clone. In order to detect a putative PG synthesis in all the clones tested, we decided to analyze the lipid content of this PG-deficient strain using 2-D TLC [47]. Compared to the negative control transformed with the void vector, the PG spot was only slightly darker after diiodine detection of the lipids extracted from the strain transformed with *pgsA* [Fig 4A]. This might explain why the results of the complementation were not definitive under our conditions. One obvious explanation is the probable toxicity due to pgsA overexpression—it is difficult to propagate this particular clone because it has a short survival period on plates after transformation. Surprisingly, although the amount of PG appeared to be the same in the clone transformed with mouse CGI-58, compared to the control, this amount was clearly lower in the clone transformed with the full-length version of plant CGI-58, and considerably lower in the clone transformed with the truncated version. MN7 was shown to have a very low amount of PG [20] and, to avoid a possible artifact in this particular strain, we re-examined the phospholipid composition, especially the amount of PG, in all the strains previously used to characterize CGI-58. Surprisingly, the results were similar in the wild-type strain, BL21(DE3), and in another phospholipid synthesis mutant, SM2-1 [Fig 4A]. In both strains there was no visual difference in the clones expressing mouse CGI-58 compared to the control; there was a lower amount of PG when the full-length version of *Arabidopsis* CGI-58 was expressed in BL21(DE3); and, again, significantly less PG in both strains when the truncated version was used [Fig 4A]. We hypothesized that the truncated version has a more dramatic effect because of the removal of the putative N-terminal peptide. Since the effects of recombinant plant CGI-58 expression appeared more dramatic in the SM2-1 strain, we decided to quantify the phospholipid content in this particular strain by LC/MS/MS. Although the content of PE was not significantly different between all the clones tested [Fig 4B], the content of PG was reduced by 40% in clones expressing the truncated version of AtCGI-58, compared to the control [Fig 4C], confirming the results obtained by TLC analysis [Fig 4A].

There could be several reasons for the decrease in PG content: either PG or the precursors of PG synthesis [48], such as CDP-diacylglycerol-glycerol-3-phosphate, are used as a substrate by plant CGI-58 to form another undetected lipid or, alternatively, CGI-58 may have a regulatory role in phospholipid synthesis. To test the hypothesis that PG could be a substrate for plant CGI-58, we synthesized labeled PG, from [^14^C]-PC, using the phospholipase D-catalyzed transphosphatidylation reaction of [^14^C]-PC and glycerol. However, under the conditions used, neither the incubation of crude extract from the BL21(DE3) strain expressing plant CGI-58 nor the incubation of purified truncated protein in the presence of [^14^C]-PG led to PG hydrolysis (data not shown), suggesting that either an acceptor of the PG moiety was missing in the enzymatic test or that PG is not a real substrate of recombinant plant CGI-58. The observation that CGI-58 may have a role in determining PG content or in homeostasis has already been described in the literature concerning mouse CGI-58 [49]. When CGI-58 expression was inhibited in adult mice by antisense oligonucleotides, there was a 10-fold increase in their hepatic PG level. Since mouse CGI-58 lacks the putative catalytic seryl residue, we could hypothesize that the effect of CGI-58 on the PG level may not be directly linked to its hydrolytic abilities, but rather to a signaling function. The fact that the amount of PG is affected by the expression of CGI-58 has already been observed *in planta* as well. An analysis of lipid profiles in plants mutated for CGI-58 showed that PG levels increased compared to the wild-type, whereas the amount of other major plant phospholipids (PC, PE, phosphatidylinositol and phosphatidylserine) was unchanged [4]. It is always possible that mammalian CGI-58s are not functional in *E*. *coli*, compared to eukaryotic expression systems and, hence, no effect is detected in this prokaryotic system. It would be interesting to measure all the enzymatic activities involved in phospholipid metabolism in *E*. *coli* in order to detect any changes due to CGI-58 expression and to restrict the metabolic route affected by its expression.

### Using a PG phenotype to test for the importance of catalytic residues

**Fig 5:**
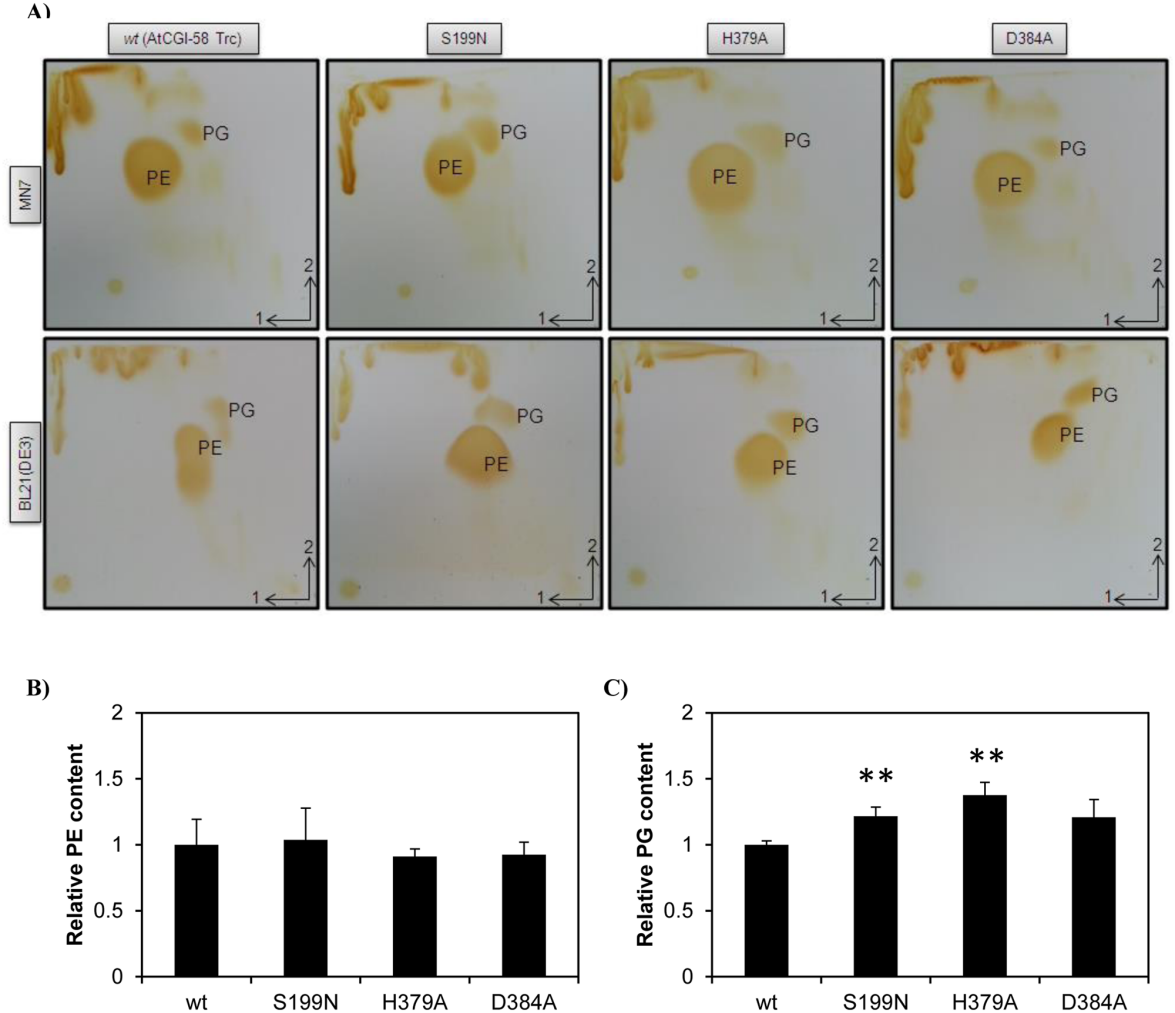
Analysis of phospholipids extracted from strains transformed with mutated versions of plant *CGI-58*. A) TLC analysis of total lipids extracted from strains MN7 and BL21(DE3). Lipids were extracted and separated as described in Fig 4. Experiments were carried out at the same time and under the same conditions and, for comparison, TLC pictures of wild-type AtCGI-58 Trc were duplicated from Fig 4. Each experiment was repeated at least twice. B) Comparison of the PE content of SM2-1 transformants determined by LC/MS/MS. Total lipids were extracted and analyzed. The quantities of PE were calculated based on a 36:2 PE standard curve and normalized to the PE content of the wild-type (AtCGI-58 Trc). Values ± SD are the mean of three independent clones. C) Comparison of the PG content of SM2-1 transformants determined by LC/MS/MS. Total lipids were extracted and analyzed. The quantities of PG were calculated based on a 28:0 PG standard curve and normalized to the PG content of the wild-type (AtCGI-58 Trc). Values ± SD are the mean of three independent clones. ** *P*<0.01, (*vs* wt).

Interestingly, the nature of the artefactual LPAAT activity of mouse CGI-58 was proven when the activity of the wild-type protein was discovered to be equivalent to that of recombinant CGI-58 with mutations in putative catalytic residues [18]. In order to prove that a catalytic activity, rather than a putative regulatory bias of plant CGI-58, was involved in the decrease of PG in *E*. *coli*, we decided to analyze the effect of a mutation of the seryl residue at position 199, using the difference in PG levels in transformants to screen other putative catalytic residues of recombinant plant CGI-58 protein. The seryl residue at position 199 is replaced in mammals by an asparaginyl and this residue is thought to be involved in the catalytic reaction [12,50]. In addition, histidyl 379 and aspartyl 384 have been identified as belonging to the HX_4_D motif, which is indispensable for glycerolipid acyltransferase catalysis [51], and a particular 3-D modeling of murine CGI-58 has placed these 3 amino acids in close proximity to one another [12,50]. In order to verify the importance of the residues in the catalytic reaction, we designed primers to change these three residues by site-directed mutagenesis in plant CGI-58 and, hence, we created S199N, D384A and H379A versions of the truncated plant CGI-58. Mutated versions of plant CGI-58 were then expressed with the pYAT7 plasmids in MN7, SM2-1 and BL21(DE3) strains. Lipids were again extracted and separated by 2D-TLC for the MN7 and BL21(DE3) strains [Fig 5A]. Mutations S199N and H379A led to a visual increase of the PG level in both strains, demonstrating that S199 and H379 residues are involved in the decrease of PG seen in strains transformed with the wild-type construct. The amount of PG in mutant D384 is quite similar to that found in the corresponding wild-types, suggesting that this residue is not involved in the catalytic reaction. Consequently, we decided to quantify the amount of phospholipids, by LC/MS/MS, in SM2-1 transformants where we had previously observed the more dramatic effects. Compared to the wild-type version of the truncated plant protein, no statistical difference in PE content could be observed among the different mutations [Fig 5B]. However, the PG content was significantly increased by 22 and 38% when the S199N and H379A versions of the protein were expressed respectively. The amount of PG was not statistically different from that of the wild-type when the D384A version was expressed. This D384 residue is not completely conserved among plant CGI-58s [S1 Fig] and it is sometimes replaced by Glutamyl, an alternate acidic residue, in *Theobroma cacao* and *Solanum lycopersicum*. It is still possible that another residue, such as D302 or D351 that is absolutely conserved in all plant CGI-58s [S1 Fig], could be the third member of the catalytic triad. We should point out that D351 has been identified [50] as the proposed catalytic residue in a 3D-modeling of murine CGI-58. Moreover, another 3-D modeling of murine CGI-58 has placed the homolog of the D384 residue on the exterior surface of the protein rather than in the internal pocket [18]. As previously suspected, S199 is involved in the catalytic reaction. It is perhaps obvious that mammalian CGI-58 harboring an asparaginyl residue at the same position as the seryl residue plays no role in the decrease of PG, as shown in Fig 4, suggesting divergent roles for plant and mammalian CGI-58. Measuring the PG content could now be used as a screening method to establish which amino acid is important for the catalytic reactions involving plant CGI-58.

## Conclusion

Although their role in lipid metabolism is demonstrated again here, plant and mammalian CGI-58 seem to have different catalytic roles, at least in regards to their involvement in the decrease of PG levels in *E*. *coli*. The catalytic activity of plant CGI-58 is still to be elucidated although we have shown for the first time that the seryl residue at position 199 and the histidyl residue at position 379 are implicated in recombinant plant CGI-58 activity. There are also several lines of evidence indicating that its activity is closely related to PG metabolism. *In planta*, the amount of PG has been shown to increase if *CGI-58* is mutated, and we show in this paper that the quantity of PG decreases in bacteria when the protein is overexpressed. Since PG is directly linked with cardiolipin synthesis, some alternative roles of plant CGI-58, involving either PG precursors or PG sequels, are currently being investigated.

## Supporting Information

S1 Fig Alignment of plant CGI-58s. Sequences were retrieved from NCBI with the protein BLAST software, using *At*CGI-58 as bait, from Mosses through to Eudicot proteins. Alignment was performed with BioEdit software and the shading threshold was set to 75% identity. *Arabidopsis thaliana* At4g24160 was retrieved from a sequenced RAFL19-16-I19 clone and the *Physcomitrella patens* sequence was reconstructed from a sequencing of ESTs BY990945.1 and BJ940330.1. All the aforementioned clones were obtained from the Riken Institute [22,23]. Accession numbers are as following: *Camelina sativa* XP_010439078.1, *Brassica rapa* XP_009137743.1, *Populus trichocarpa* XP_002307698.2, *Theobroma cacao* XP_007017700.1, *Nelumbo nucifera* XP_010271460.1, *Eucalyptus grandis* XP_010061022.1, *Musa acuminata subsp*. *malaccensis* XP_009418733.1, *Sesamum indicum* XP_011083599.1, *Solanum lycopersicum* XP_004243145.1, *Nicotiana sylvestris* XP_009794253.1, *Citrus sinensis* XP_006473603.1, *Ricinus communis* XP_002510485.1, *Phoenix dactylifera* XP_008813831.1, *Medicago truncatula* XP_003603733.1, *Glycine max* XP_006577977.1, *Fragaria vesca subsp*. *vesca* XP_004291822.1, *Malus domestica* XP_008347352.1, *Prunus persica* XP_007222711.1, *Picea glauca* BT117776.1, *Cucumis sativus* XP_004152662.1, *Amborella trichopoda* XP_006854859.1, *Beta vulgaris subsp*. *vulgaris* XP_010670751.1, *Zea mays* ACF79200.1, *Oryza sativa Japonica Group* NP_001063697.2, *Brachypodium distachyon* XP_003578450.1, *Sorghum bicolor* XP_002460538.1. Positions of mutated residues S199, H379, and D384 of *Arabidopsis thaliana* CGI-58 are indicated by arrows.
(TIF)

## Abbreviations

ABTS: 2,2'-azino-bis(3-ethylbenzothiazoline-6-sulphonic acid)
ATGL: Adipose triglyceride lipase
CGI-58: Comparative Gene Identification-58
DOPC: 1,2-Dioleoyl-*sn*-glycero-3-phosphocholine
DOPG: 1,2-Dioleoyl-*sn*-glycero-3-phosphoglycerol
DPPC: 1,2-Dipalmitoyl-*sn*-glycero-3-phosphocholine
DPPG: 1,2-Dipalmitoyl-*sn*-glycero-3-phosphoglycerol
ICT1: Increased Copper Tolerance 1
IPTG: Isopropyl β-D-1-thiogalactopyranoside
LPAAT: Lysophosphatidic acid acylCoA acyltransferase
LPGAT: Lysophosphatidylglycerol acylCoA acyltransferase
LPA: Lysophosphatidic acid
LPG: Lysophosphatidylglycerol
PA: Phosphatidic acid
PC: Phosphatidylcholine
PE: Phosphatidylethanolamine
PG: Phosphatidylglycerol
pgsA: Phosphatidylglycerophosphate synthase
PLA_2_: Phospholipase A_2_
PLD: Phospholipase D
plsC: 1-acyl-*sn*-glycerol-3-phosphate acyltransferase
POPG: 1-palmitoyl-2-oleoyl-*sn*-glycero-3-phosphoglycerol
T7RP: T7 RNA polymerase
TAG: Triacylglycerol
TLL: *Thermomyces lanuginosus* lipase
