# Supplementary figures and images for: Reassessing the Potential Activities of Plant CGI-58 Protein
Source: PLoS One. 2016 Jan 8;11(1):e0145806. doi: 10.1371/journal.pone.0145806 (PMC4706320; doi:10.1371/journal.pone.0145806)

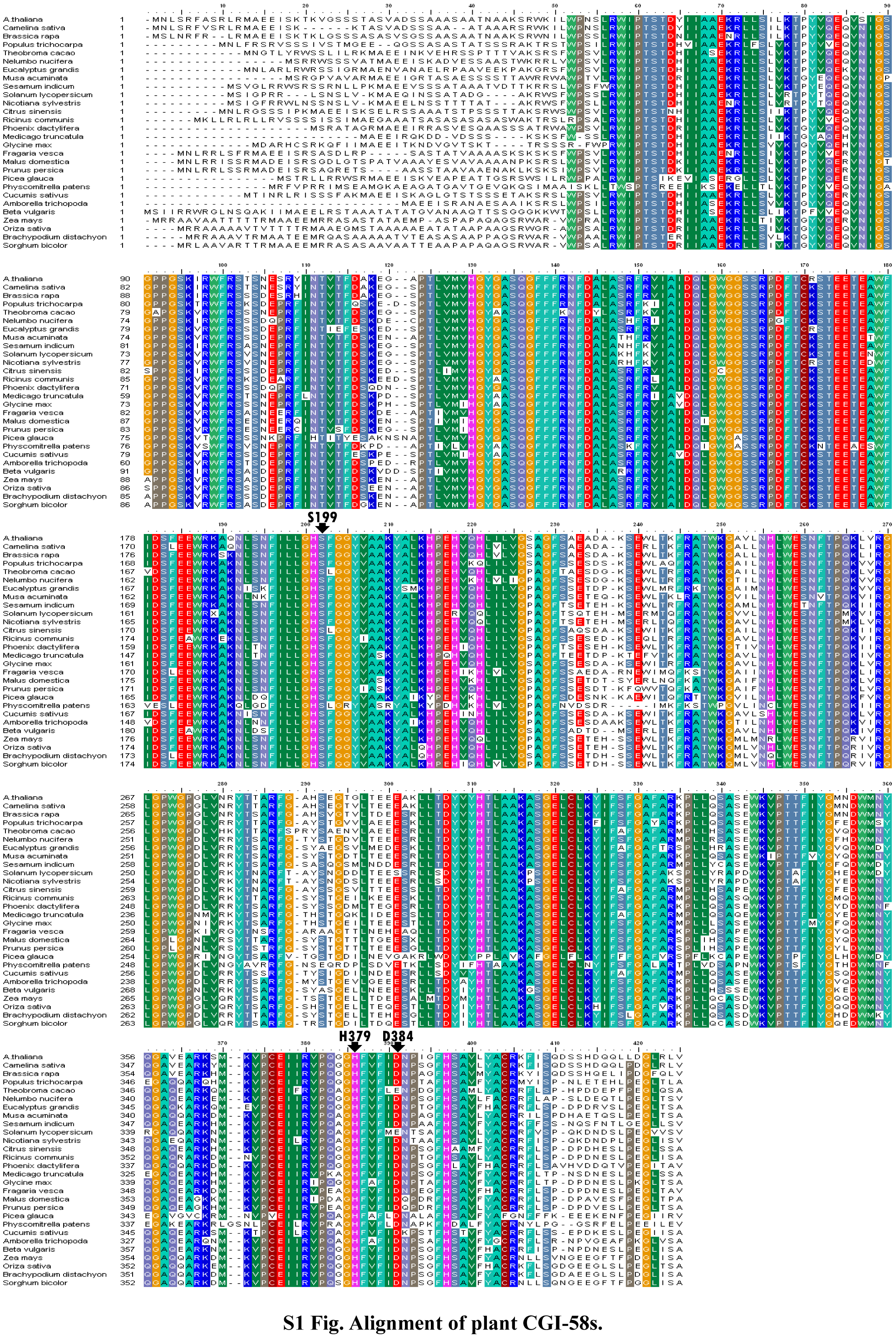

Supplement: S1 Fig — Sequences were retrieved from NCBI with the protein BLAST software, using AtCGI-58 as bait, from Mosses through to Eudicot proteins. Alignment was performed with BioEdit software and the shading threshold was set to 75% identity. Arabidopsis thaliana At4g24160 was retrieved from a sequenced RAFL19-16-I19 clone and the Physcomitrella patens sequence was reconstructed from a sequencing of ESTs BY990945.1 and BJ940330.1. All the aforementioned clones were obtained from the Riken Institute [22,23]. Accession numbers are as following: Camelina sativa XP_010439078.1, Brassica rapa XP_009137743.1, Populus trichocarpa XP_002307698.2, Theobroma cacao XP_007017700.1, Nelumbo nucifera XP_010271460.1, Eucalyptus grandis XP_010061022.1, Musa acuminata subsp. malaccensis XP_009418733.1, Sesamum indicum XP_011083599.1, Solanum lycopersicum XP_004243145.1, Nicotiana sylvestris XP_009794253.1, Citrus sinensis XP_006473603.1, Ricinus communis XP_002510485.1, Phoenix dactylifera XP_008813831.1, Medicago truncatula XP_003603733.1, Glycine max XP_006577977.1, Fragaria vesca subsp. vesca XP_004291822.1, Malus domestica XP_008347352.1, Prunus persica XP_007222711.1, Picea glauca BT117776.1, Cucumis sativus XP_004152662.1, Amborella trichopoda XP_006854859.1, Beta vulgaris subsp. vulgaris XP_010670751.1, Zea mays ACF79200.1, Oryza sativa Japonica Group NP_001063697.2, Brachypodium distachyon XP_003578450.1, Sorghum bicolor XP_002460538.1. Positions of mutated residues S199, H379, and D384 of Arabidopsis thaliana CGI-58 are indicated by arrows. (TIF) [file pone.0145806.s001.tif]
